# Positive Modulation Effect of 8-Week Consumption of *Kaempferia parviflora* on Health-Related Physical Fitness and Oxidative Status in Healthy Elderly Volunteers

**DOI:** 10.1155/2012/732816

**Published:** 2012-07-31

**Authors:** Jintanaporn Wattanathorn, Supaporn Muchimapura, Terdthai Tong-Un, Narisara Saenghong, Wipawee Thukhum-Mee, Bungorn Sripanidkulchai

**Affiliations:** ^1^Department of Physiology, Faculty of Medicine, Khon Kaen University, Khon Kaen 40002, Thailand; ^2^Integrative Complementary Alternative Medicine Research and Development Group, Khon Kaen University, Khon Kaen 40002, Thailand; ^3^Center for Research and Development of Herbal Medicine, Khon Kaen University, Khon Kaen 40002, Thailand

## Abstract

Health-related physical fitness declines as the age advances. Oxidative stress is reported to contribute the crucial role on this phenomenon. This condition is also enhanced by antioxidant. Therefore, we aimed to determine the effect of *Kaempferia parviflora*, a plant reputed for antifatigue, longevity promotion, and antioxidant effects, on health-related quality physical fitness and oxidative status of the healthy elderly volunteers. Total 45 subjects had been randomized to receive placebo or *K. parviflora* extract at doses of 25 or 90 mg once daily for 8 weeks. They were determined baseline data of physical performance using 30 sec chair stand test, hand grip test, 6 min walk test, and tandem test. Serum oxidative stress markers including malondialdehde (MDA) level and the activities of superoxide dismutase (SOD), catalase (CAT), and glutathione peroxidase (GSH-Px) were also assayed. All assessments were performed every 4 weeks throughout the 8-week study period. The results showed that *K. parviflora* increased performance in 30-second chair stand test and 6 min walk test together with the increased all scavenger enzymes activities and the decreased MDA level. Therefore, *K. parviflora* can enhance physical fitness partly via the decreased oxidative stress. In conclusion, *K. parviflora* is the potential health supplement for elderly. However, further study is required.

## 1. Introduction

Health-related physical fitness is defined as fitness related to some aspect of health. It is regarded as a major marker of health status at any age. It comprises of 4 main domains including strength and endurance of skeletal muscles, joint flexibility, body composition, and cardiorespiratory endurance [1]. Physical fitness is varied depending on the age. It has been reported that physical fitness achieves the peak performance during late teens and begins a slow decline in their early 20s. Therefore, the definition of physical fitness must be defined with consideration for an individual's age. It is defined as a physical condition that allows an individual to work without becoming overly fatigued, perform daily chores, and have enough energy left over to engage in leisure activities in younger person, whereas it is defined as the physical condition that allows an individual to conduct daily activity without becoming exhausted or tired. Thus, the decreased health-related physical fitness produces great impact on quality of life and disability condition of the elderly. Therefore, the ultimate goal in ageing society nowadays is to maintain the health-related physical fitness of the elderly.

Recent findings showed that age-related physical decline might be related to oxidative damage perpetrated by free radicals [2]. Free radicals disrupt the homeostasis of biological systems by damaging their major constituent molecules, leading eventually to cell death [3, 4]. It has been reported that oxidative damage may play a crucial role in the decline of functional activity in human skeletal muscle with normal aging [5, 6]. Recent findings showed that plasma antioxidant concentrations correlate positively with physical performance and strength. Higher dietary intakes of most antioxidants, especially vitamin C, are associated with higher skeletal muscular strength in elderly persons [7]. In addition to the muscle strength, oxidative stress also plays an important role on cardiopulmonary performance, which can be assessed via 6 min walk test [8].

Since the health-related quality of life produces great impact on quality of life and it is under the influence of many factors, various strategies have been implemented to enhance and maintain the health-related quality of life. Among various strategies, phytomedicine or herbal therapy, which has been long-term used in traditional folklore to treat various ailments and to restore physical fitness [9], has gained much attention.

*K. parviflora* Wall. ex Baker or Krachai Dam is belonging to the family of Zingiberaceae. It has been long term used in Thai traditional medicine for treating various ailments including allergy, fatigue, sexual dysfunction, and ulcer. In addition, it is also used as longevity promoting substance and as nerve tonic. Recent findings showed that *K. parviflora *rhizomes extract contained numerous flavonoids [10], which was previously reported to possess antioxidant activity, neuroprotective, and cognitive-enhancing effects [11]. Based on the antifatigue and antioxidant effect of *K. parviflora*, we hypothesized that *K. parviflora* might enhance the physical fitness and oxidative status in healthy elderly. To elucidate this issue, we aimed to determine the effect of 8-week consumption of *K. parviflora* extract on health-related physical fitness and on oxidative stress status of healthy elderly volunteers.

## 2. Materials and Methods

### 2.1. Subjects

A total 45, healthy elderly volunteers were initially recruited to take part in a randomized trial designed to investigate the effects of an 8-week consumption of *K. parviflora* on health-related physical fitness. Subjects were volunteers who were older than 60 years, healthy, and without history of cardiovascular diseases, respiratory diseases, neuropsychological diseases, head injury, diabetes, cancer, alcohol addiction, and smokers of more than 10 pieces per day. Any persons taking prescribed and nonprescribed drugs or nutraceutical compounds influencing the function of the nervous system were also excluded. All participants were also requested and agreed to abstain from caffeine-containing products, throughout each study day, and alcohol for a minimum of 12 h prior to the test sessions. This study was approved by the Khon Kaen University Ethics Committee of Human Research.

Prior to the participation, each volunteer had signed an informed consent form and completed a medical health questionnaire. All recruited subjects were screened for healthy status again by the physician. In addition, the blood was also collected for the determination of oxidative stress markers.

### 2.2. **Kaempferia parviflora ** Preparation

A standardized extract of *K. parviflora* was prepared by the Center for Research and Development of Herbal Health Product, Faculty of Pharmaceutical Sciences, Khon Kaen University. All *K. parviflora* used in this study was obtained from Loei Province. The plant was authenticated and kept as voucher specimen at Faculty of Pharmaceutical Sciences, Khon Kaen University. Standardization and conformity of the extract is assured by strict in-process controls during manufacture and complete analytical control of the resulting dry extract. A-day capsule contained a specialized rhizome extract containing 5,7 dimethoxyflavone (2.1%), 5,7,4′-trimethoxyflavone (3.1%), and 3,5,7,3′,4′-pentamethoxyflavone (2.3%). Each *K. parviflora* capsule contained crude extract of *K. parviflora* at doses of 25 and 90 mg.

Placebo tablets were manufactured using the same pharmaceutical excipient and replicated the active in appearance, odor, and texture. Packaging and randomization was performed by Integrative Complementary and Alternative Medicine Research and Development Group, Khon Kaen University, the study coordinator.

### 2.3. Procedures and Intervention

In this study, we determined the health-related physical fitness by using modified method of Fanò et al., which focused on muscular strength and cardiopulmonary endurance [6]. In addition to the domain just mentioned, we also focused on the postural control because it produced great influence on risk to fall of the elderly. Therefore, our tests were consisted of 30-second chair stand test, hand grip strength test, 6-minute walk test, and tandem test. The 30-second chair stand test was used to assess the strength of skeletal muscle especially muscle of the lower extremity whereas hand grip strength test was used to assess the strength of muscle of the upper extremity, especially the hand muscle. The cardio-pulmonary endurance was performed via 6-minute walk test, whereas the postural control was assessed using tandem test. Subjects were assessed the physical fitness with the same sequence in all assessments. The health-related physical fitness and oxidative stress markers were assessed every 4 weeks throughout the experimental period.

The code for study allocation was only broken when the last participant completed the entire followup. Staffs involved in the collection of the study's endpoints were instructed to follow a rigorous protocol and not to discuss any issues related to the use of medication. The review of compliance with medication and side-effects was performed independently by the investigators, who were also blinded to group allocation. Adverse effects were assessed during every study visit. Subjects were requested to call the study center if they experienced any medical problems during the 8-week study period.

### 2.4. Health-Related Physical Fitness Assessment

To assess the health-related physical fitness in the elderly, we used the battery test as follows.

**30-Second Chair Stand Test**

This test was used to evaluate lower-body muscular strength. According to this test, the number of times within 30 second that an individual can rise to a full stand from a seated position with back straight and feet flat on the floor, without pushing off with the arms, was recorded.

**Handgrip Strength Test**

This test was the upper-body muscular strength by using a digital dynamometer. Subjects performed (alternately with both hands) the test twice allowing a 1-minute rest period between measures. The best value of 2 trials for each hand was chosen, and the average of both hands was registered.

**6-Minute Walk Test**

This test involves the determination of the maximum distance (meters) that can be walked in 6 min along a 45.7 meters rectangular course. It reflects the cardio-pulmonary endurance.

**Tandem Stance Test**

This test was performed with both eyes opened and with eye closed while one foot placed in front of the other foot when both feet touching each other. Standing duration without swaying was recorded.

### 2.5. Determination of Oxidative Stress Markers

Fasting venous blood sample was collected in all subjects and care was taken. Serum was separated and analyzed for oxidative stress parameters including the level of malondialdehyde (MDA) and the activities of superoxide dismutase (SOD), catalase (CAT), and glutathione peroxidase (GSH-Px). MDA was measured by thiobarbituric acid reactive substances assay (TBRAS) method [12]. SOD activity was measured using the xanthine/xanthine oxidase reaction as a source of substrate (superoxide) and reduced nitroblue tetrazolium as an indicator of superoxide [13]. The activity of CAT was assayed based on the decomposition of substrate H_2_O_2_, which was monitored via spectrophotometrically at 340 nm for 5 minutes [14], whereas the activity of GSH-Px was performed using t-buthylhydroperoxide as the substrate [15].

### 2.6. Statistical Analysis

All data are expressed as mean ± S.E.M. Between-group comparisons and the comparison between baseline data and the changes observed at various time points of physical fitness and biochemical parameters were performed using analysis of variance (ANOVA). Post hoc, Dunnett test was used after using one-way analysis of variance. Statistical significance was regarded at *P* value <0.05.

## 3. Results

### 3.1. Demographic Data of Subjects

The baseline data about demographic data of subjects in all groups were shown in Table 1. No significant differences of all parameters among various groups were observed.

### 3.2. Effect of *K. parviflora* on Health-Related Physical Fitness

Effects of various doses of *K. parviflora* on various parameters indicating physical fitness were shown in Table 2. It was found that subjects who consumed *K. parviflora* at dose of 90 mg/day significantly increased 30-second chair stand test (*P* value <0.05; compared to baseline data). In addition, it was found that subjects who consumed the extract at dose of 90 mg/may increase 6 min walk test (*P* value <0.05 all; compared to either baseline or placebo treated group). However, no other significant effects were observed.

### 3.3. Effect of *K. parviflora* on Oxidative Stress Markers

**Figure 1:**
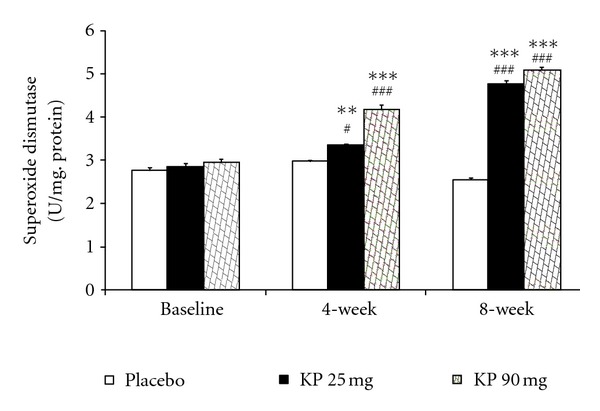
Effect of various doses of *K. parviflora* on level of superoxide dismutase in serum. Data were present as mean ± SEM (*n* = 15/group). ^∗∗,∗∗∗^ *P* value <0.01; 0.001 compared with placebo group, respectively. ^#,###^ *P* value <0.05; 0.01 compared to baseline, respectively.

**Figure 2:**
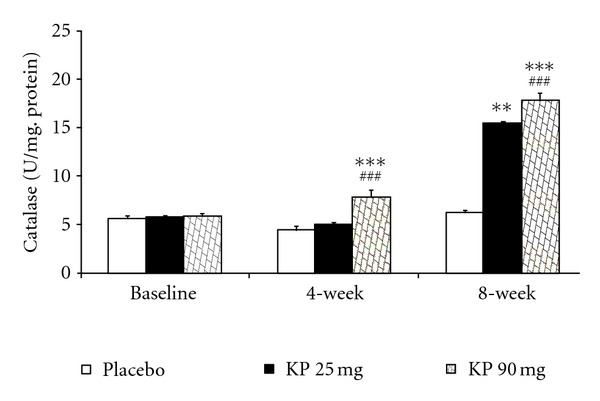
Effect of various doses of *K. parviflora* on level of catalase in serum. Data were present as mean ± SEM (*n* = 15/group). ^∗∗,∗∗∗^ *P* value <0.01; 0.001 compared with placebo group, respectively. ^###^ *P* value <0.001 compared to baseline.

**Figure 3:**
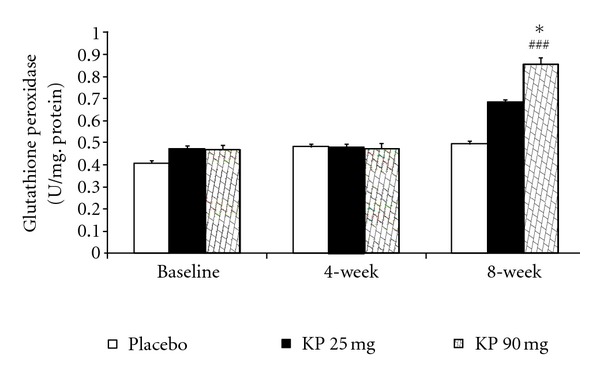
Effect of various doses of *K. parviflora* on level of glutathione peroxidase in serum. Data were present as mean ± SEM (*n* = 15/group). *P value <0.05 compared with placebo group. ^##^ *P* value <0.01 compared to baseline.

**Figure 4:**
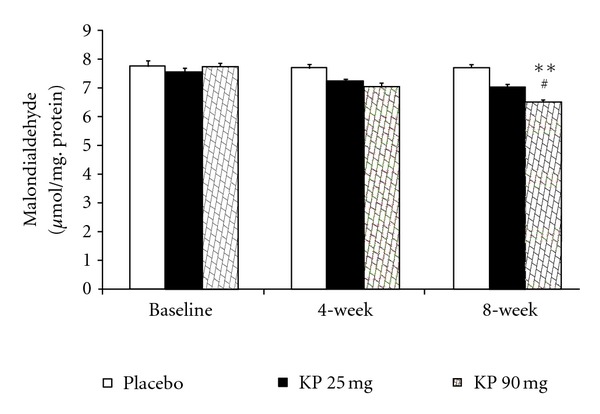
Effect of various doses of *K. parviflora* on level of malondialdehyde (MDA) in serum. Data were present as mean ± SEM (*n* = 15/group). **P value <0.01 compared with placebo group. ^#^ *P* value <0.05 compared to baseline.

The effect of *K. parviflora* on various oxidative stress markers including superoxide dismutase (SOD), catalase (CAT), and glutathione peroxidase (GSH-Px) activities and the level of malondialdehyde (MDA) in serum were shown in Figures 1–4. Our data showed that subjects who consumed *K. parviflora* extract at dose of 25 mg/day showed the significant increase in SOD activity at 4-week (*P* value <0.01 compared to placebo-treated group; *P* value <0.05 compared to baseline data) and 8-week period (*P* value <0.001 all; both compared to placebo treated group and compared to baseline data). However, subjects who consumed the low dose of extract failed to show significant changes of CAT and GSH-Px activities and MDA level at 4-week intervention period. It was found that at 4-week study period, subjects who consumed *K. parviflora* extract at dose of 90 mg showed the significant elevation of SOD activity (*P* value <0.05; compared to baseline data and *P* value <0.001 compared to placebo-treated group). The significant elevation of CAT activity was also observed (*P* value <0.001; compared to placebo treated group), whereas no significant changes of GSH-Px activity and MDA level were not observed at this duration. When the consumption period was increased further to 8 weeks, it was found that subjects who consumed *K. parviflora* at dose of 90 mg showed the significant elevation of SOD (*P* value <0.001 all; both compared to placebo treated group and compared to baseline data), CAT (*P* value <0.001 all; both compared to placebo-treated group and compared to baseline data), and GSH-Px activities (*P* value <0.05 compared to placebo treated group and *P* value <0.01 compared to baseline data). In addition, the decreased MDA level was also observed (*P* value <0.01 compared to placebo treated group; *P* value <0.05 compared to baseline data).

## 4. Discussion

This study has clearly revealed that *K. parviflora* significantly enhanced the performance in 30-second chair stand test and 6 min walk test which reflect the enhanced strength of muscle of lower extremities and the enhanced cardiopulmonary endurance together with the improved oxidative stress status.

Current trend in the dramatically increased elderly population enhances the importance of sustaining physical fitness of this group of population. It has been reported that as the age advances, the physical fitness declines. Both muscle area and fiber numbers are decreased since the fourth decade [16]. A parallel decrease in muscle strength (knee extension) also occurs with the decrease in muscle mass. In addition to the decreased muscle mass, the decreased muscle efficiency such as decreased oxygen uptake [17] and the decreased muscle mitochondria ATP production [18], the decreased ratio between type I and type II [19], are also observed. All factors mentioned earlier are also essential for the strength of muscle limb and aerobic or cardiopulmonary endurance.

A recent finding has shown that the enhanced blood flow plays the crucial role on the metabolic implications, which in turn influenced on the functional capacity of the muscle [20], and oxidative stress interferes ATP production of mitochondria [21]. Since we also found the enhanced antioxidant enzymes activities and the decreased MDA level in this study, we suggested that the enhanced performance of muscle of lower extremities in subject following 8-week consumption of *K. parviflora* at dose of 90 mg/day might be associated with the enhanced blood flow [22, 23] and the decreased oxidative stress [24] of this medicinal plant.

Our data showed the improved muscle strength only in the lower extremities while no significant changes of muscle of extremities were observed. Since muscle of the lower extremities contained more type I muscle fiber, a muscle with high vascular supply, than the muscle of the upper extremities, we did suggest that the effect of *K. parviflora* selectively depended on types of muscle and the main principal action of *K. parviflora* might be associated with its vasodilation effect resulting in the enhanced blood flow especially in muscle of lower extremities.

It has been clearly demonstrated that 6 min walk test is a valid and reliable measurement of physical endurance in elderly [25]. The aerobic endurance, which reflect the function, of cardiopulmonary function, is under the influence of antioxidant. Substance possessing antioxidant has been previously reported to enhance oxygen utilization [26]. Theoretically, improved oxygen usage could improve aerobic endurance performance. Recent finding also showed that flavonoid could increase muscle oxidative capacity and endurance in mice [27]. Therefore, *K. parviflora*, which contained flavonoid and possessed antioxidant effect, might enhance oxygen usage and oxidative capacity and resulting in the increased performance in aerobic endurance manifesting by enhanced capability in 6-minute walk test.

**Figure 5:**
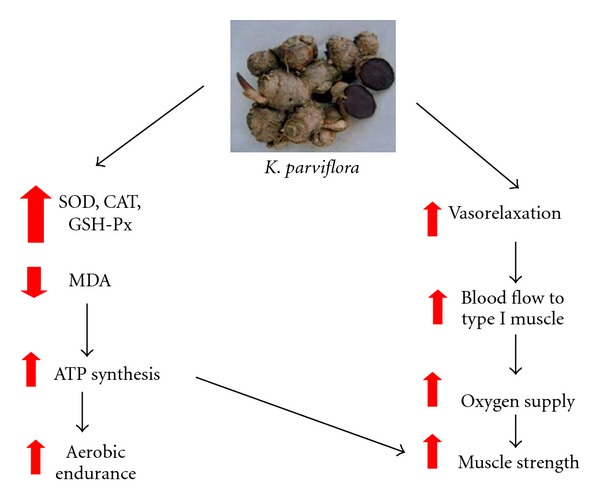
Schematic diagram illustrated the possible action of *K. parviflora* on muscle strength of lower extremities and aerobic endurance.

Taken all data together, *K. parviflora* could enhance blood flow to muscle, enhance oxygen utilization, and decrease oxidative stress which in turn enhanced ATP production capacity of mitochondria and resulted in the increased muscle strength especially in lower extremities and enhanced aerobic endurance as shown in Figure 5. In addition, unpublished data of our colleagues also showed that subchronic toxicity of *K. parviflora* extract is safe up to 500 mg/kg. Therefore, the safety range of this extract is quite wide and may be possible to develop as food supplement for elderly.

## 5. Conclusions

*K. parviflora* or Thai ginseng is the potential food supplement to enhance muscle strength and aerobic endurance, the important components of health-related physical fitness. Therefore, it may also improve health quality of life and decrease risk to fall in the elderly. However, further study is required.

## Figures and Tables

**Table 1:** Demographic data of subjects (*n* = 15/group).

General characteristic	Placebo	KP25	KP90
			62.66 ± 6.33
Age (years)	64.2 ± 6.95	61.53 ± 6.39	*F*(0.05,2, 42) = 0.6236,
			*P* = 0.5409

			7.33 ± 2.96
Education (years)	7.73 ± 2.65	7.53 ± 2.82	*F*(0.05,2, 42) = 0.0754,
			*P* = 0.9275

			91.26 ± 4.31
Full-scale IQ	90.6 ± 7.66	89.2 ± 63.55	*F*(0.05,2, 42) = 0.4160,
			*P* = 0.6624

			120.20 ± 5.94
Systolic blood pressure (mmHg)	122.26 ± 8.22	121.20 ± 6.63	*F*(0.05,2, 42) = 0.3270,
			*P* = 0.7229

			84.80 ± 6.47
Diastolic blood pressure (mmHg)	81.53 ± 6.84	83.26 ± 7.64	*F*(0.05,2, 42) = 0.8165,
			*P* = 0.4489

			23.04 ± 1.67
Body mass index	21.92 ± 2.06	23.27 ± 1.34	*F*(0.05,2, 42) = 2.6763,
			*P* = 0.50805

Data were present as mean ± SEM.

**Table 2:** Effect of various doses of *K. parviflora* on health related physical fitness.

Measured parameters	Group	Pre-dose	1 month	2 month
	Placebo	24.53 + 2.55	24.33 + 2.28	24.33 + 2.46
Grip strength (Rt) (kg)	KP25	25.06 + 3.01	25 + 2.97	24.86 + 3.18
	KP90	23.93 + 3.30	24.6 + 3.13	24.8 + 3.14

	Placebo	21.06 + 1.83	21.33 + 1.58	21.2 + 1.56
Grip strength (Lt) (kg)	KP25	22.06 + 1.86	21.66 + 1.5	21.26 + 1.48
	KP90	20.86 + 2.72	21.6 + 2.02	21.6 + 1.84

	Placebo	19.13 + 2.79	19.26 + 1.43	18.93 + 1.70
30-second chair stand test. (sec)	KP25	18.33 + 2.58	19 + 2.77	20 + 3.11
	KP90	18.6 + 2.52	19.6 + 2.13	20.66 + 2.28^#^

	Placebo	567.33 + 33.52	598.73 + 31.57	571.26 + 32.05
6 min. walk test (m.)	KP25	571.26 + 33.68	570.33 + 38.32	575.53 + 36.04
	KP90	572.8 + 32.65	575.46 + 34.29	601.26 + 33.70^∗#^

	Placebo	164.8 ± 12.34	163.06 ± 10.35	165.06 ± 9.80
Tandem test (Opened Eye, Right leg is in front) (sec)	KP25	161.8 ± 11.16	164.06 ± 9.63	162.26 ± 8.93
	KP90	164 ± 10.50	166.6 ± 6.81	168.46 + 6.90

	Placebo	112.33 ± 11.00	110.66 ± 10.01	109 + 10.20
Tandem test (Opened Eye, Left leg is in front) (sec)	KP25	111.93 ± 7.77	112.33 ± 11.39	111.8 + 10.16
	KP90	108.2 ± 11.32	109.33 ± 13.62	110.46 + 13.31

	Placebo	33.8 ± 9.22	30.8 ± 10.74	31.66 + 10.41
Tandem test (Closed Eye, Right leg is in front) (sec)	KP25	31.86 ± 10.12	32.6 ± 7.44	32.73 + 7.67
	KP90	31.26 ± 11.09	31.86 ± 9.33	33.4 + 8.94

	Placebo	18.8 + 3.60	19.86 + 5.01	21.2 + 4.57
Tandem test (Closed Eye, Left leg is in front) (sec)	KP25	20.93 + 3.41	21.33 + 3.79	21.26 + 3.19
	KP90	20.46 + 4.24	21.26 + 4.58	22.06 + 3.93

Data were present as mean ± SEM (*n* = 15/group).

*P value <0.05 compared to placebo, ^#^ *P* value <0.05 compared to baseline.
